# GacA is essential for Group A *S* *treptococcus* and defines a new class of monomeric dTDP‐4‐dehydrorhamnose reductases (RmlD)

**DOI:** 10.1111/mmi.13169

**Published:** 2015-10-01

**Authors:** Samantha L. van der Beek, Yoann Le Breton, Andrew T. Ferenbach, Robert N. Chapman, Daan M. F. van Aalten, Iva Navratilova, Geert‐Jan Boons, Kevin S. McIver, Nina M. van Sorge, Helge C. Dorfmueller

**Affiliations:** ^1^ University Medical Center UtrechtMedical MicrobiologyHeidelberglaan 1003584 CXUtrechtThe Netherlands; ^2^ Department of Cell Biology and Molecular GeneticsMaryland Pathogen Research InstituteUniversity of Maryland3124 Biosciences Research BuildingCollege ParkMD 20742USA; ^3^ Division of Molecular MicrobiologyUniversity of DundeeSchool of Life SciencesDow StreetDD1 5EHDundeeUK; ^4^ Complex Carbohydrate Research CenterDepartment of ChemistryThe University of Georgia315 Riverbend RoadAthensUSA; ^5^ Division of Biological Chemistry and Drug DiscoveryUniversity of DundeeSchool of Life SciencesDow StreetDD1 5EHDundeeUK; ^6^ Rutherford Appleton LaboratoryResearch Complex at HarwellOX11 0FADidcotUK

## Abstract

The sugar nucleotide dTDP‐L‐rhamnose is critical for the biosynthesis of the Group A Carbohydrate, the molecular signature and virulence determinant of the human pathogen Group A *S* *treptococcus* (GAS). The final step of the four‐step dTDP‐L‐rhamnose biosynthesis pathway is catalyzed by dTDP‐4‐dehydrorhamnose reductases (RmlD). RmlD from the Gram‐negative bacterium *S* *almonella* is the only structurally characterized family member and requires metal‐dependent homo‐dimerization for enzymatic activity. Using a biochemical and structural biology approach, we demonstrate that the only RmlD homologue from GAS, previously renamed GacA, functions in a novel monomeric manner. Sequence analysis of 213 Gram‐negative and Gram‐positive RmlD homologues predicts that enzymes from all Gram‐positive species lack a dimerization motif and function as monomers. The enzymatic function of GacA was confirmed through heterologous expression of *gac* *A* in a *S*. *mutans rml* *D* knockout, which restored attenuated growth and aberrant cell division. Finally, analysis of a saturated mutant GAS library using Tn‐sequencing and generation of a conditional‐expression mutant identified *gac* *A* as an essential gene for GAS. In conclusion, GacA is an essential monomeric enzyme in GAS and representative of monomeric RmlD enzymes in Gram‐positive bacteria and a subset of Gram‐negative bacteria. These results will help future screens for novel inhibitors of dTDP‐L‐rhamnose biosynthesis.

## Introduction

The cell wall of Gram‐positive bacteria is an intricate network of peptidoglycan, proteins and secondary cell wall polymers (SCWPs) that are covalently linked to peptidoglycan. Teichoic or teichuronic acids are typical and well‐studied SCWP in Gram‐positive bacteria and play an important role in normal cell function and infection (Weidenmaier and Peschel, 2008). Many β‐hemolytic streptococcal species appear to lack expression of typical teichoic or teichuronic acid structures (Sutcliffe *et al*., 2008; Caliot *et al*., 2012) and instead express a rhamnose‐rich polymer, which comprises approximately half of the cell wall mass (McCarty, 1952). Historically, expression of these evolutionary conserved glycans underlies classification of β‐hemolytic streptococci in Lancefield groups (A, B, C, G …) (Lancefield, 1933), a feature that is still applied in contemporary rapid test kits to diagnose streptococcal infections.

*Streptococcus pyogenes*, also referred to as Group A *Streptococcus* (GAS), is a β‐hemolytic human‐restricted pathogen and ranks in the top 10 of infection‐related causes of mortality worldwide (Carapetis *et al*., 2005). GAS is the causative agent of a wide spectrum of clinical disease, including common localized infections (∼ 700 million cases per year worldwide) and approximately 1.8 million cases of severe disease (Carapetis *et al*., 2005), including necrotizing fasciitis, streptococcal toxic shock syndrome and post‐infectious streptococcal sequelae, i.e. acute rheumatic fever and rheumatic heart disease. A better understanding of GAS pathogenesis and development of new drugs and protective vaccines is crucial. GAS expresses a characteristic SCWP known as Lancefield Group A Antigen or Group A Carbohydrate (GAC) (Lancefield, 1933). The GAC structure consists of a polyrhamnose core decorated with alternating immunodominant *N*‐acetylglucosamine (GlcNAc) side‐chains (Coligan *et al*., 1978). Recently, van Sorge *et al*. (2014) identified the gene cluster responsible for GAC biosynthesis and demonstrated that the GAC GlcNAc side‐chain contributes to GAS virulence.

In contrast to detailed insights into the biosynthesis of classical SCWP like teichoic acids, information regarding the biosynthesis of rhamnose‐rich polysaccharides GAC is limited. The production of dTDP‐L‐rhamnose is critical for GAC biosynthesis but also more broadly for the viability or virulence of other medically important bacteria including *Mycobacterium spp*. (Ma *et al*., 2002), *Pseudomonas spp*. (Engels *et al*., 1985) and *Enterococcus faecalis* (Teng *et al*., 2005). dTDP‐L‐rhamnose is synthesized from α‐glucose‐1‐phosphate through a four‐step enzymatic process catalyzed by the enzymes RmlA‐D (Kornfeld and Glaser, 1961; Pazur and Shuey, 1961). Structural analysis of RmlA‐D from *Pseudomonas aeruginosa* (RmlA, Blankenfeldt *et al*., 2000), *Streptococcus suis* (RmlB, Beis *et al*., 2003 and RlmC, Dong *et al*., 2003) and *Salmonella enterica* (*Se*) (RmlD, Blankenfeldt *et al*., 2002), have provided valuable insights into the mechanism of action for these enzymes, including requirement for Mg^2+^‐dependent dimerization in the case of RmlD. In general, RmlD enzymes are members of the large short‐chain dehydro‐genases/reductases (SDR) superfamily, which act on a wide family of substrates and commonly form homo‐dimeric or multimeric complexes (Kavanagh *et al*., 2008).

In GAS, homologues of RmlA, B and C that catalyze the first steps of the dTDP‐rhamnose biosynthesis pathway can be identified through bioinformatics and are clustered on the genome. The GAS RmlD homologue appears to be encoded by the *gacA* gene, which is annotated as a dTDP‐4‐dehydrorhamnose reductase, but experimental data supporting this function is currently lacking. The goal of this study was to identify the function and structure of the *gacA* gene product through biochemistry, structural biology and bacterial genetics. We show that *gacA* is an essential gene of GAS that encodes a metal‐independent dTDP‐4‐dehydrorhamnose reductase representative of a new class of monomeric RmlD enzymes.

## Results and discussion

### GacA encodes a functional metal‐independent dTDP‐4‐dehydrorhamnose reductase (RmlD)

Bioinformatics analysis suggests that *gacA* encodes a dTDP‐4‐dehydrorhamnose reductase, an enzyme that catalyzes the final step in the production of dTDP‐L‐rhamnose (Giraud and Naismith, 2000). In contrast to the *rmlD* genes in other species like *Shigella flexneri* (Macpherson *et al*., 1994) and *Streptococcus pneumoniae* serotype 19F (Morona *et al*., 1997), the only GAS *rmlD* homologue *gacA* is not part of an *rmlABCD* rhamnose biosynthesis operon. Instead, *gacA* is located at the beginning of the recently identified GAC gene cluster and hence named *gacA* (van Sorge *et al*., 2014). A similar split genomic architecture of the rhamnose biosynthesis genes *rmlA‐C* and *rmlD* was previously observed in *Streptococcus mutans* (*S. mutans*), a cariogenic Gram‐positive bacterium (Tsukioka *et al*., 1997).

**Figure 1:**
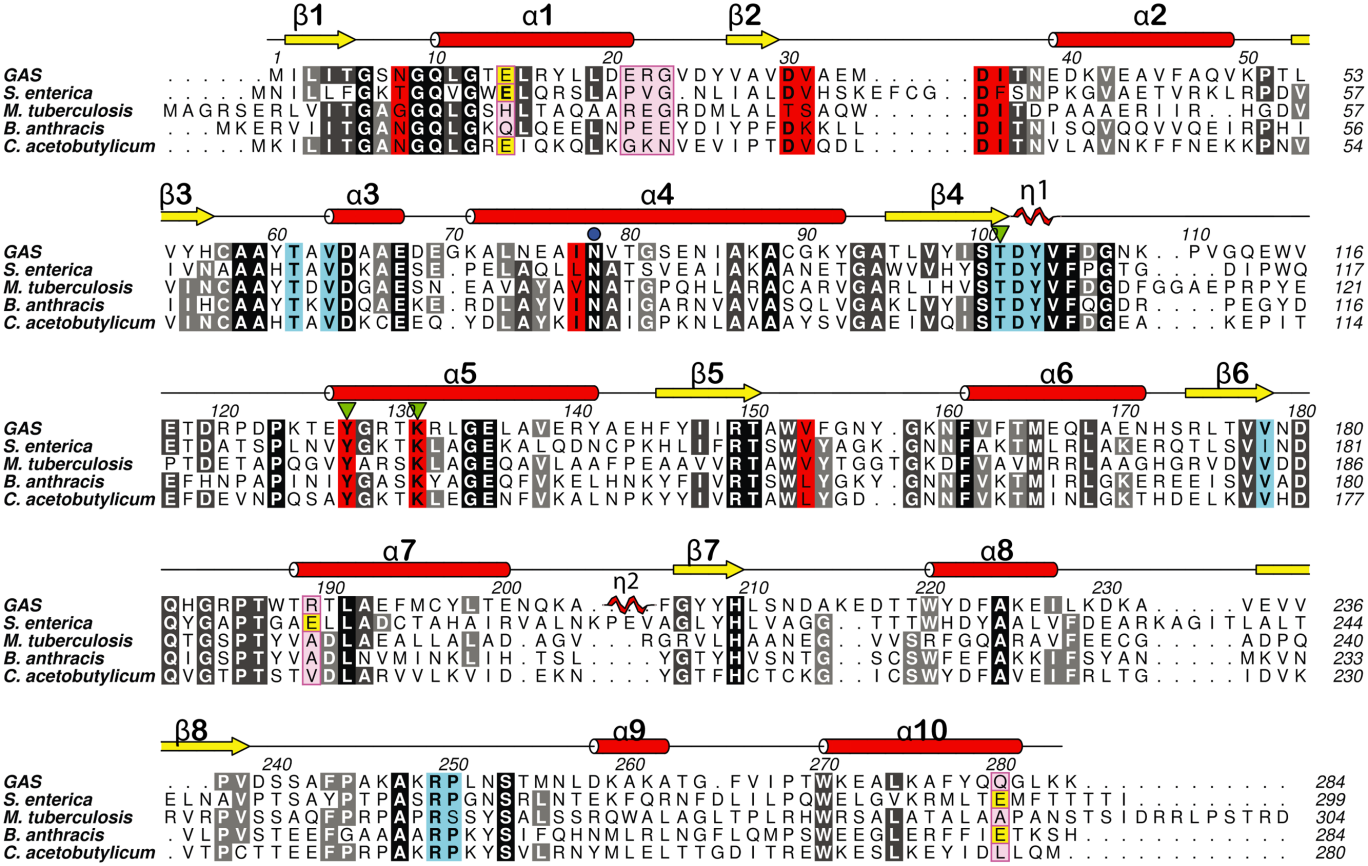
Sequence alignment for GacA and RmlD homologues. Sequence alignment of GAS GacA with RmlD from *S* *almonella enterica* serovar Typhimurium (Blankenfeldt *et al*., 2002), *M* *ycobacterium tuberculosis* (accession number WP_009938025), the hypothetical RmlD homologues from *B* *acillus anthracis* str. Ames (accession number NP_843703) and *C* *lostridium acetobutylicum* (*a*ccession number WP_010965612). Conserved residues are colored in black (> 80%) and gray (60–80%). Substrate binding site residues are colored in turquois and cofactor binding site residues in red. Secondary structure elements from GacA are indicated and labeled with red α‐helices and yellow β‐strands. Mg^2+^‐binding site residues from *Se*RmlD are colored in yellow (E1–E3) and the corresponding non‐conserved residues in the other species are colored in magenta (R1–R3). The terminal residues of the α1‐helix motif are highlighted with magenta boxes. N78 is indicated with a blue dot, and the catalytic triad is indicated with green triangles.

**Figure 2:**
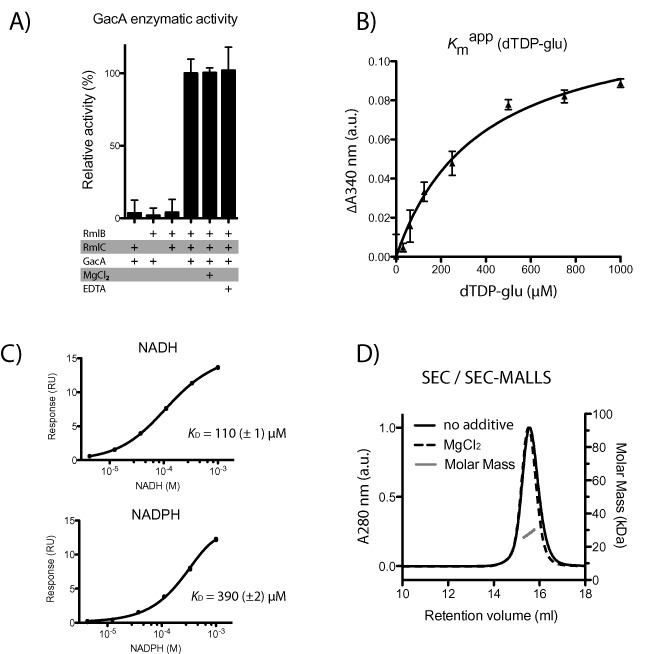
GacA is a functional metal‐independent monomeric dTDP‐4‐dehydrorhamnose reductase. A. Biochemical confirmation that GacA is a functional and metal‐independent dTDP‐4‐dehydrorhamnose reductase. Read‐out of GacA enzyme activity is change in absorbance at 340 nm, indicating oxidation of the GacA cofactor NADPH to NADP ^+^, was observed. GacA is only active in the presence of coupled enzymes *Sp*RmlB and *Sp*RmlC, which provide the substrate for GacA. GacA metal dependence was investigated in presence and absence of 10 mM MgCl_2_ and 10 mM EDTA. B. GacA Michaelis‐Menten kinetics were determined for dTDP‐glucose (*K*_m_ value of 370 μM). The second substrate NADPH was present in excess. C. Surface plasmon resonance equilibrium fit for the binding of NADPH and NADH to GacA. NADPH and NADH were injected over a concentration range of 4.1 μM to 1,000 μM. Equilibrium affinity fit values are shown in the bottom panel of the figure. D. Size‐exclusion chromatography (SEC) and SEC‐MALLS of recombinant purified GacA in presence (dashed line) and absence (solid line) of 10 mM MgCl_2_. All samples reveal the same retention volume, corresponding to a calculated average molecular mass of 27.5 kDa (blue line) demonstrating GacA is a monomer.

We set out to investigate the potential function of GacA as a dTDP‐4‐dehydrorhamnose reductase. We cloned and expressed full‐length GacA fused to a cleavable GST‐His_6_ tag. The GAS GacA protein sequence is 36% identical to the *Salmonella enterica* serovar Typhimurium RmlD protein (*Se*RmlD) (Fig. 1), the only reported RmlD structure (Blankenfeldt *et al*., 2002). To confirm the enzymatic activity of GacA *in vitro*, we also cloned, expressed and purified the putative RmlB and RmlC GAS homologues, as the RmlD substrate dTDP‐4‐dehydrorhamnose is not commercially available. In this biochemical assay, oxidation of NADPH is a read‐out for GacA activity. No activity was observed with any combination of two enzymes (Fig. 2A). However, we observed significant oxidation of NADPH when all three enzymes were present, suggesting that GacA is indeed acting as a dTDP‐4‐dehydrorhamnose reductase (Fig. 2A). As mentioned above, the enzyme activity of *Se*RmlD was shown to be metal dependent (Blankenfeldt *et al*., 2002). Similarly, RmlD from *Mycobacterium tuberculosis* (*Mtb*RmlD) was assayed in presence of 10 mM MgCl_2_, despite the lack of biochemical evidence that the enzyme requires a divalent cation for enzymatic activity (Wang *et al*., 2011). To investigate the metal‐dependent activity of GacA, we performed the assay in presence and absence of 10 mM MgCl_2_ and/or 10 mM EDTA. No significant changes in enzymatic activity were observed for GacA (Fig. 2A), demonstrating that unlike *Se*RmlD (Blankenfeldt *et al*., 2002), the proper positioning of the cofactor is not dependent on a metal‐ion (Graninger *et al*., 1999). The GacA enzyme activity assay was subsequently performed in absence of MgCl_2_.

Next, we studied Michaelis‐Menten kinetics of GacA. The *K*_m_^app^ value for dTDP‐α‐glucose was determined to be 370 μM (Fig. 2B), in agreement to the 110 μM *K*_m_ value determined for the *Se*RmlD homologue (Blankenfeldt *et al*., 2002). Using surface plasmon resonance, we investigated the binding affinity of NADPH and NADH to GacA, which were previously shown to be functional cofactors for RmlD enzymes (Blankenfeldt *et al*., 2002). Both substrates bind to GacA with binding affinities of *K*_D_ = 390 ± 2 μM (NADPH) and *K*_D_ = 110 μM ± 1 μM (NADH) (Fig. 2C).

### GacA is active as a monomer

Many SDR family members, including *Se*RmlD, require dimerization or oligomerization to be functional (Blankenfeldt *et al*., 2002; Kavanagh *et al*., 2008). Results from our functional enzymatic assay demonstrated that GacA did not require metal for its activity, suggesting that it might be functional as a monomer. GacA was analyzed by size‐exclusion chromatography (SEC) and SEC‐MALLS and a molecular mass of the elution peak was calculated. The calculated mass of GacA is 27.5 kDa (± 2.4 kDa) (Fig. 2D). This is in good agreement with the theoretical calculated monomeric mass of 32 kDa of the GacA polypeptide sequence and represents the first monomeric RmlD enzyme. We subsequently investigated the effect of Mg^2+^‐ions on protein size. The purified protein was incubated with and without 10 mM MgCl_2_ overnight at 4°C and analyzed via SEC in the corresponding buffers (Fig. 2D). Both protein samples show identical elution volumes at 15.8 ml, with an average calculated molecular mass of 27.5 kDa, indicating that no mass change/dimerization occurs in presence of Mg^2+^ (Fig. 2D).

### *M. tuberculosis* RmlD inhibitors inhibit recombinant GacA

Wang *et al*. (2011) have identified a series of *Mtb*RmlD inhibitors by virtual screening using the crystal structure of *Se*RmlD. The sequence identity between *Mtb*RmlD and *Se*RmlD is 31%, whereas the sequence identity between GacA and *Mtb*RmlD is 36% (Fig. 1). We assessed the inhibitory effect of two of the four previously described inhibitors using enzyme kinetics. Our experiments show that both compounds inhibit the activity of recombinant GacA with an IC_50_ of approx. 2 μM for compound 3 and approx. 10 μM for compound 2. These data are in good agreement to the *Mtb*RmlD inhibition with corresponding IC_50_ values of 0.9 μM and 15 μM, respectively (Wang *et al*., 2011).

### GacA crystal structure reveals novel monomeric RmlD form with a conserved catalytic triad

To unravel why GacA functions as a monomer, we employed X‐ray crystallography. Crystals diffracted routinely below 1.2 Å and were subjected to synchrotron data collection. Results were compared with the four GacA (RmlD) homologues that have been deposited in the Protein Data Bank (PDB) (Bernstein *et al*., 1977) (Supp. Fig. S1): The Gram‐negative *Se*RmlD (pdb entry 1kbz, 1n2s, 1kc1, 1kc3) (Blankenfeldt *et al*., 2002), *Clostridium acetobutylicum* RmlD (*Ca*RmlD; pdb entry 1vl0), the Gram‐positive *Bacillus anthracis* RmlD (*Ba*RmlD; pdb entry 3sc6) and the archaea *Sulfolobus tokodaii* RmlD (*St*RmlD; pdb entry 2ggs).

**Figure 3:**
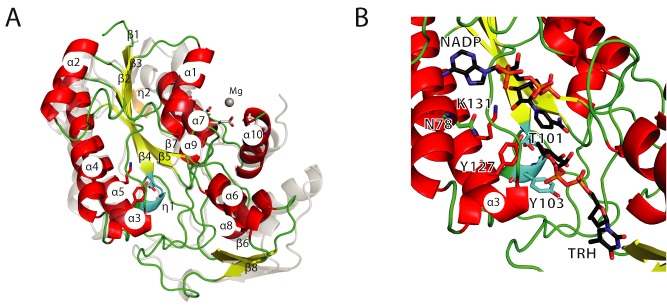
Structural insights into a monomeric Gram‐positive RmlD enzyme. A. Comparison of the GacA secondary structure elements with *Se*RmlD [PDB entry 1KBZ (Blankenfeldt *et al*., 2002)], a dTDP‐4‐dehydrorhamnose reductase. The structures are shown in a cartoon representation. GacA is colored with red helices and yellow strands; *Se*RmlD is colored in transparent gray. Secondary structure elements are labeled according to Fig. 1. *Se*RmlD Mg^2+^‐binding site is shown with the three glutamic acids (gray sticks) co‐ordinating the Mg^2+^‐ion (gray sphere). B. Active site view with cartoon and stick representation of GacA in complex with superimposed ligands NADPH and dTDP‐L‐rhamnose (TRH) from the ternary *Se*RmlD complex [PDB entry 1KC3 (Blankenfeldt *et al*., 2002)]. The conserved catalytic residues T101, Y127 and K131 (GacA) and N78 are shown as sticks, color‐coded according to Fig. 1.

Consistent with our SEC and activity studies, GacA crystallized as a monomer. The overall structure of the GacA monomer aligns well with all four RmlD structures in the database, with Cα rmsd of 1.4 Å (apo, *Se*RmlD, 1kbz, Fig. 3A), 1.1 Å (NADPH complex, *Ba*RmlD, 3sc6) and 1.0 Å (NADH complex, *Ca*RmlD, 1vl0) and 1.4 Å (NADPH complex, *St*RmlD, 2ggs, Supp. Fig. S1). GacA contains the typical α‐helical/β‐sheet arrangement known as Rossmann fold present in SDR domains (Fig. 3A). In agreement with the well‐characterized *Se*RmlD (Blankenfeldt *et al*., 2002), the GacA β‐sheet is formed of six β‐strands, in the order 213457 (Fig. 3A). This β‐sheet is flanked by three and four α‐helices on either side, respectively. The previously described prominent kink in α‐helix 4, a characteristic structural feature in SDR enzymes, is also present in GacA and is caused by the conserved Asn78 (Asn81 in *Se*RmlD) (Figs. 1 and 3B). The second 3_10_‐helix η2 observed in *Se*RmlD (Blankenfeldt *et al*., 2002) is missing in GacA due to a shorter loop connecting α‐helix α7 and β‐strand β7 (Figs. 1 and 3A). The α‐helices α1‐5, α7 and α9 form the cofactor binding site, whereas α‐helices α6, α8 and α10 form the substrate‐binding domain (Fig. 3A and B). In comparison with *Se*RmlD, α8 from GacA lacks two α‐helical turns, due to a deletion of eight amino acids in GacA (Figs. 1 and 3A). Furthermore, we calculated the hydrodynamic radius (Stokes radius) of the soluble GacA and the crystallized monomer. The soluble GacA has a R_h_ = 26 Å (± 0.3%), in good agreement with the R_h_ calculated from the obtained crystal structure using HYDROPRO (R_h_ = 26 Å) (Ortega *et al*., 2011). No symmetry‐related GacA molecule forms protein–protein interactions via α1 and α10 as observed in *Se*RmlD (Fig. 3A) (Blankenfeldt *et al*., 2002). The GacA crystal structure confirms that GacA defines a new class of monomeric RmlD enzymes that do not require metal for enzymatic activity (Fig. 3A).

We superimposed the ternary *Se*RmlD complex (Blankenfeldt *et al*., 2002) onto the GacA crystal structure to investigate active site sequence conservation (Figs. 1 and 3B). The GacA crystal structure is in the ‘open’ conformation, allowing binding of the cofactor NADPH and the acceptor substrate dTDP‐4‐dehydrorhamnose. All active site residues are conserved with the *Se*RmlD (Fig. 1, green triangles). The catalytic triad identified for *Se*RmlD (Blankenfeldt *et al*., 2002), consisting of T104, Y128 and K132, occupies identical conformations in GacA (T101, Y127 and K131, Figs. 1 and 3B), suggesting that GacA is a functional monomeric RmlD homologue using a conserved catalytic mechanism.

### RmlD enzymes from Gram‐positive bacteria are monomers due to lack of a conserved RmlD dimerization motif

**Figure 4:**
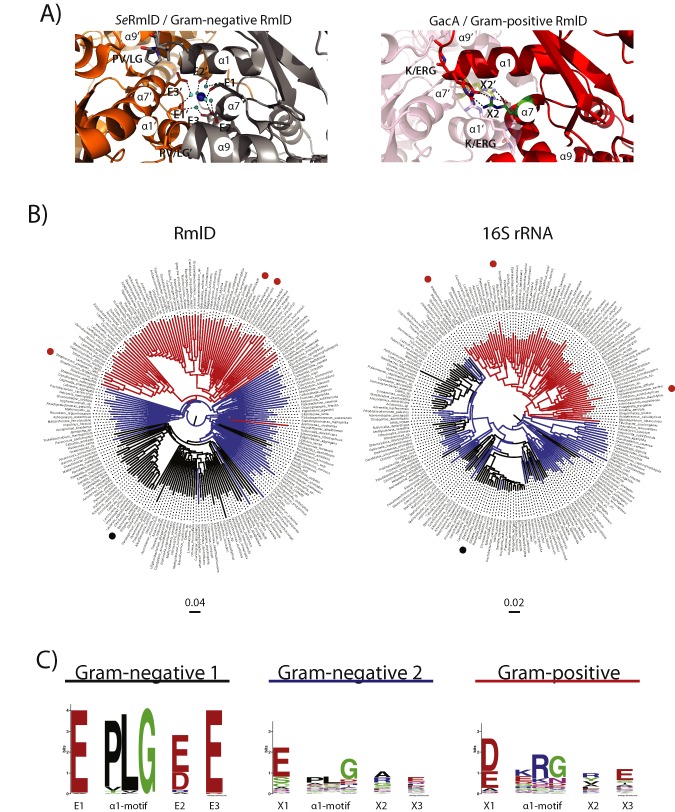
GacA represents a new class of monomeric RmlD enzymes. A. Structural insight in dimerization interface of RmlD reveals steric hindrances that would prevent homo‐dimerization in Gram‐positive RmlD enzymes. Left: View of the *Se*RmlD homo‐dimer Mg^2+^‐binding site, representing Gram‐negative RmlD enzymes (Blankenfeldt *et al*., 2002). The E1‐E/D2‐E3 motif co‐ordinates the Mg^2+^‐ion. The α1‐helix contains the PLG motif, which allows dimerization, as the P introduces a turn into the α1‐helix. Right: Hypothetical model of a GacA homo‐dimer based on the *Se*RmlD homo‐dimer (red and transparent cartoon representation). The K/ERG motif prevents dimerization, as it extends the α1‐helix by half a turn, which would clash with the α7′‐helix of the second molecule. The X2 side‐chain (R189) points into the Mg^2+^‐binding pocket and prevents dimerization (salmon and red arginine side‐chains). Large, basic or hydrophobic residues, mainly R or Y, replace the E2 motif in Gram‐positive RmlD homologues. A salt bridge between GacA E21 from the ERG motif and X2 (R189) stabilizes the tertiary structure of the α1‐ and α7‐helices, which is located at the corresponding position of the Gram‐negative Mg^2+^‐binding site (green and yellow side‐chains). B. Neighbor‐Joining trees of 213 RmlD orthologous sequences (left) and their corresponding 16S rRNA sequences (right). Gram‐positive bacteria are colored in red, Gram‐negative that contain the conserved dimerization sequence logo E1‐PLG‐E/D2‐E3 are colored in black (‘Gram‐negative 1’) and Gram‐negative bacteria that lack one or both of the dimerization criteria are colored in blue (‘Gram‐negative 2’). *S* *treptococcus pyogenes* (GAS), *C* *lostridium acetolyticum* and *B* *acillus anthracis* are marked with red dots, *S* *almonella enterica* with a black dot. C. Left: Sequence logo of dimerization interface for Gram‐negative RmlD enzymes. 135 (putative) orthologous RmlD sequences from Gram‐negative bacteria (Supp. Table S1) were analyzed for their E1, E2, E3 and α1‐helix motifs, which is critical for Mg^2+^‐binding and dimerization as described by Blankenfeldt *et al*. (Blankenfeldt *et al*., 2002). Seventy RmlD homologues contain the ‘Gram‐negative 1’ motif E‐PLG‐E/D‐E (Supp. Table S1). Sixty‐five RmlD homologues are lacking these conserved residues (‘Gram‐negative 2’) and are therefore expected to function as monomers (Supp. Table S2). Right: Seventy‐eight RmlD sequences from (putative) Gram‐positive bacteria (Supp. Table S3) were analyzed for the same motifs. The Gram‐positive RmlD homologues lack a distinctive motif and therefore lack the ability to co‐ordinate a divalent metal ion. The ‘Gram‐negative 1’ α1‐helix PLG‐motif is replaced in Gram‐positive RmlD sequences by a K/ERG motif.

To investigate the molecular basis for the novel monomeric RmlD form, we focused on the amino acids at the *Se*RmlD dimerization interface. From the published *Se*RmlD structure (Blankenfeldt *et al*., 2002), there appear to be two critical parameters for *Se*RmlD dimerization: (i) the three glutamate residues E15, E190 and E292 at the *Se*RmlD dimerization interface that help co‐ordinate the Mg^2+^‐ion (Figs. 1 and 4A) and (ii) a shortened α1‐helix caused by a proline residue (P22, *Se*RmlD) that allows binding of the second *Se*RmlD monomer via tight protein–protein interactions (Fig. 4A). To experimentally confirm the contribution of these two parameters to *Se*RmlD dimerization, we cloned, expressed and purified the wild‐type RmlD enzyme from *S. enterica* and designed a triple‐mutant (3M), in which the putative key residues for *Se*RmlD dimerization (PVG, E, E) are replaced with the corresponding residues in the monomeric GacA (ERGV, R, Q). SEC analysis revealed that the *Se*RmlD triple mutant is a monomeric protein, identical to GacA (Supp. Fig. S2A), whereas wild‐type *Se*RmlD runs at a shorter retention time, in agreement to its higher dimeric mass (Blankenfeldt *et al*., 2002). Furthermore, EDTA treatment of wild‐type *Se*RmlD does not affect the retention time (Supp. Fig. S2A), suggesting that the removal of the Mg^2+^‐ion does not disrupt dimerization. The peak fractions were analyzed using fingerprint mass‐spectrometry and contained the correct protein.

GacA lacks both of the confirmed critical dimerization parameters as it does not contain all conserved negatively charged E residues and has an extended α1‐helix due to the ERG motif that occupies the putative Mg^2+^‐binding pocket (Fig. 4A). Interestingly, the GacA structure reveals that a salt bridge is introduced (E21 and R189), stabilizing the tertiary structure of the α1‐ and α7‐helices, which could contribute to the fact that GacA functions as a monomer (Fig. 4A). We have modeled an artificial GacA dimer (Fig. 4A), which further reveals structural features that support why GacA functions as a monomer. The large, basic R22 at the end of the α1‐helix points with its side‐chain into the modeled second monomer. Furthermore, the extended α1‐helix would clash with the α7‐helix of the second GacA molecule (Fig. 4A). These features are incompatible with homo‐dimerization, but most likely stabilize the monomeric enzyme.

We analyzed whether the lack of structural dimerization features is unique to GacA or are present in RmlD enzymes from other species. Using two independent psi‐BLAST runs, one starting with *Se*RmlD as a representative of dimeric RmlD enzymes and the other starting with GacA as a representative of monomeric RmlD enzymes, we identified 213 bacterial (putative) RmlD homologues, including 78 from Gram‐positive and 135 from Gram‐negative species (Suppl. Tables S1–S3). All full‐length RmlD protein sequences as well as the corresponding 16S rRNA DNA sequences of the same 213 species were aligned and used to build bootstrapped Neighbor‐Joining trees (Fig. 4B). Interestingly, RmlD sequences from all Gram‐positive bacteria, except *Desulfitibacter alkalitolerans*, clustered separately from the RmlD sequences of Gram‐negative bacteria. We aimed to determine whether the observed phylogenetic split was associated with sequence differences at the dimerization interface. Therefore, we aligned the 135 Gram‐negative and 78 Gram‐positive RmlD sequences corresponding to the critical negatively charged amino acids and the PLG sequence identified at the *Se*RmlD dimerization interface (Supp. Tables S1–S3). Interestingly, the RmlD sequences from Gram‐negative species could be divided in two groups. The group named ‘Gram‐negative 1’ contained the fully conserved dimerization site represented as an E1‐PLG‐E/D2‐E3 motif (Fig. 4C ‘Gram‐negative 1’) and similar to the previously described metal‐dependent dimeric *Se*RmlD (Supp. Table S1). This strongly suggests that RmlD proteins from this subset of Gram‐negative species form dimers. The remaining ∼ 45% of Gram‐negative RmlD enzymes lacked one or both of the critical dimerization parameters (‘Gram‐negative 2’; Fig. 4C, Supp. Table S2) and likely function as monomers similar to GacA. Superimposing the presence/absence of the dimerization motif on the 16S rRNA tree suggests that Gram‐negative bacteria have gained or lost the RmlD dimerization motif multiple times during evolution (Fig. 4B). Strikingly, RmlD sequences from all Gram‐positive species also lacked a conserved ‘Gram‐negative 1’ dimerization motif (Fig. 4C ‘Gram‐negative 1’ and ‘Gram‐positive’). For example, most Gram‐positive RmlD enzymes replaced the negatively charged glutamate at E2 with a large positively charged arginine or aromatic tyrosine substitute (X2 in Fig. 4C) and the position at E3 is also substituted in Gram‐positive bacteria compared with ‘Group 1’ Gram‐negative species (X3 in Fig. 4C). In addition, RmlD in Gram‐positive bacteria have an extended α1‐helix by half a turn due to lack of the PLG motif. This is illustrated in the artificial GacA dimer that we modeled based on the *Se*RmlD homo‐dimer (Fig. 4A) as well as in the deposited RmlD crystal structures from the Gram‐positive species *C. acetobutylicum Ca*RmlD (1VL0.pdb, Suppl. Fig. S1) and *B. anthracis Ba*RmlD (3SC6.pdb, Suppl. Fig. S1). These enzymes have not been biochemically characterized; however, both proteins did not crystallize as dimers. Furthermore, these two enzymes are similar to the GacA structure and primary sequence (Fig. 1) containing large residues at the end of the α1‐helix and missing the three conserved negatively charged E1‐E/D2‐E3 residues to accommodate a metal ion, hindering the *Se*RmlD typical dimerization (Supp. Fig. S1).

Overall, we confirmed that RmlD dimerization requires specific structural features that are absent in GacA. Additionally, comprehensive sequence analysis suggests that GacA is representative of a new class of monomeric RmlD enzymes that is present in all Gram‐positive species and a subset of Gram‐negative species.

### GacA is an essential gene for GAS during growth in rich medium

**Figure 5:**
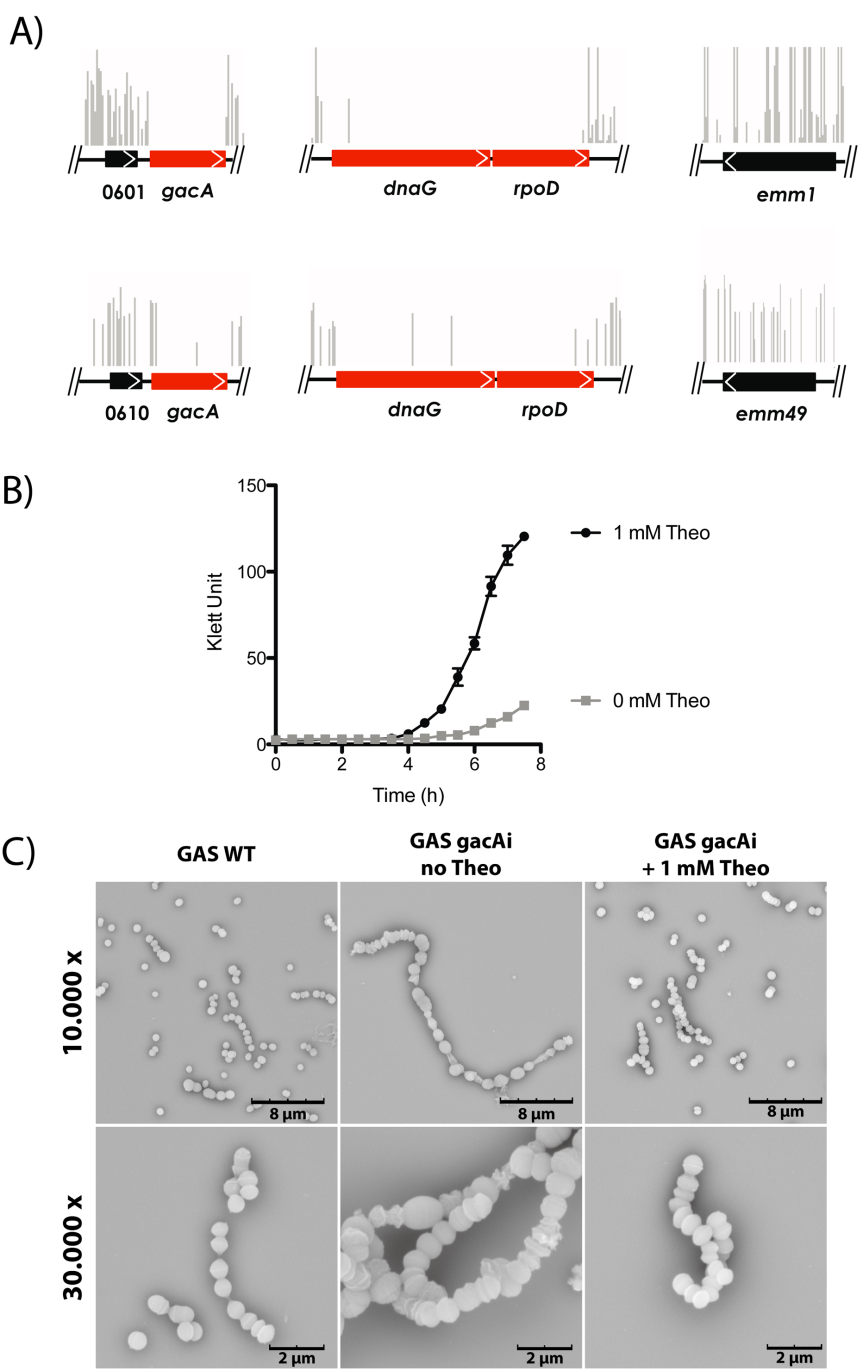
GacA is essential for GAS growth *in vitro*. A. Essentiality of *gacA* in GAS 5448 and NZ131 as determined by Tn‐seq. Complex *Krmit* transposon libraries were generated in the GAS strains 5448 and NZ131 and analyzed during growth in rich medium (THY) at 37°C. Tn‐seq analyses mapped the location and relative abundance of *K* *rmit* insertions (vertical lines) to the relevant GAS genome sequences. Using a Bayesian statistical analysis, *gac* *A* was determined to be essential (red arrows) based on limited transposon insertions (vertical lines) similar to the known essential genes *dna* *G* and *rpo* *D*. Insertions for non‐essential genes Spy_0601/0610 and *emm* from each strain are shown for comparison. B. Conditional interference with GacA expression results in severe growth attenuation of GAS. Growth parameters as followed by Klett measurements of the GAS 5448 gacAi mutant in THY rich medium in the presence (1 mM, black circles, GacA expression) or absence (gray squares, no GacA expression) of theophylline. C. Representative SEM images of GAS wild‐type strain and the generated riboswitch strain gacAi in absence and presence of 1 mM of theophylline (Theo).

To confirm the function of GacA in GAS, we attempted to generate mutants in GAS by plasmid insertion. However, we were unable to obtain mutants on multiple attempts, suggesting that *gacA* is essential for GAS (van Sorge *et al*., 2014). Additional proof for potential essentiality of *gacA* was investigated as part of a larger screen for essential genes in GAS using the mariner transposon Krmit (Le Breton *et al*., 2015). Saturated mutant libraries were produced and analyzed by Tn‐seq to identify the insertion sites within each mutant pool that survive growth in THY rich medium at 37°C. Chromosomal position and abundance of Tn‐seq reads were mapped to the *gacA* genome sequence and a Bayesian statistical analysis was performed to identify regions with limited *Krmit* insertions compared with surrounding sequences indicative of gene essentiality (Tables 1 and 2). For known essential genes *dnaG* and *rpoD* < 100 insertions per kB are observed, whereas 10‐ to 50‐fold more insertions are observed for non‐essential control genes M5005_Spy_0601 and *emm1* (Fig. 5A–F, Tables 1 and 2). Insertions for *gacA* demonstrate that *gacA* is indeed essential in the GAS strains 5448 (M1T1) and NZ131 (M49) when growing in rich media. These data are in agreement with a previous study conducted on *Mycobacterium smegmatis* (Ma *et al*., 2002), where *rmlD* was shown to be essential for mycobacterial growth. To validate *gacA* essentiality in an independent manner, we employed a previously published conditionally lethal approach that takes advantage of a theophylline‐sensitive synthetic riboswitch functional in GAS (Le Breton *et al*., 2015). In the presence of theophylline, which results in expression of *gacA*, the GAS gacAi strain was normally viable, whereas lack of theophylline significantly compromised growth of the bacteria (Fig. 5B). Visual inspection of the inducible *gacA* mutant bacteria (without theophylline) using scanning electron microscopy (SEM) indeed shows aberrant cell morphology, which defects in cell separation resulting in long chains and aberrant septum placement resulting in irregularly shaped cocci (Fig. 5C). These data underpin the critical role of rhamnose production in GAS physiology.

**Table 1:** Bayesian analysis of Tn‐seq data from GAS 5448 and NZ131

Gene^a^	GAS 5448			GAS NZ131		
	Locus^b^	Zbar^c^	Score^d^	Locus^e^	Zbar^c^	Score^d^
	Spy0601	0	NE	Spy0610	0	NE
*gacA*	Spy0602	1	E	Spy0611	1	E
*dnaG*	Spy0599	1	E	Spy0608	1	E
*rpoD*	Spy0600	1	E	Spy0609	1	E
*emm*	Spy1719	0	NE	Spy1671	0.57	NE

a When available, gene name is provided.

b Spy numbers from the MGAS5005 genome.

c Essentiality values as determined by the Bayesian analysis. Genes with a Z value over 0.992 is classified as essential, genes with Z value under 0.03 are non‐essential.

d Essentiality score. E, essential; NE, non‐essential.

e Spy numbers for the M49 NZ131 GAS genome sequence.

**Table 2:** Insertion analysis of Tn‐seq data from GAS 5448

Locus	Gene	Total number of insertions per gene	Gene size (kb)	Total insertions per gene per kb
Spy0599	*dnaG*	86	1.812	47.46
Spy0600	*rpoD*	86	1.107	77.69
Spy0601		409	0.336	1217.26
Spy0602	*gacA*	85	0.852	99.77
Spy1719	*emm1*	7877	1.452	5424.93
Average		5297	0.851	6224.44

### GAS GacA can functionally replace *S*. *mutans* RmlD

**Figure 6:**
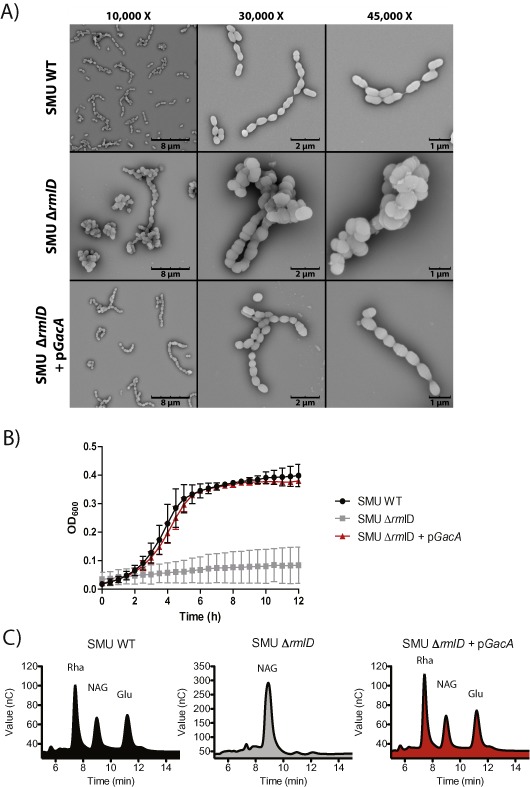
GacA functionally replaces *S*. *mutans* RmlD *in vivo.* A. Representative SEM images of *S*. *mutans* Xc (SMU) wild‐type (WT), SMUΔ*rmlD* and SMUΔ*rmlD* + p*GacA*. Cells of the wild‐type strain appear as short chains with division in a single plane, whereas the Δ*rml* *D* mutant forms clumps with long chains of swollen cocci and aberrant multi‐directional cell division. This aberrant morphology is almost completely restored by introduction of GAS *gac* *A* on an expression plasmid. B. Representative growth curves (mean ± SD, *n* = 3) of SMU WT (black circles), SMU Δ*rmlD* (grey squares) and SMU Δ*rmlD* + p*GacA* (red triangles) complemented strains cultured at 37°C without 5% CO_2_ for 12 h. (C) Analysis of monosaccharide composition of SMU WT (black), SMU Δ*rmlD* (grey) and SMU Δ*rmlD* + p*GacA* (red) by chromatography. Rha, rhamnose; NAG, GlcNAc; Glu, glucose.

To confirm the role of GacA in dTDP‐L‐rhamnose production in live bacteria, we made use of heterologous expression. It was previously shown that classical targeted disruption of *rmlD* in *S. mutans* (*Sm*RmlD) is feasible (Tsukioka *et al*., 1997; Nakano and Ooshima, 2009). Rhamnose is incorporated in the serotyping cell wall‐anchored carbohydrate composed of rhamnose decorated with a glucose side‐chain (Nakano and Ooshima, 2009). Consequently, disruption of *rmlD* results in complete loss of rhamnose and glucose from the cell wall but also significantly attenuates growth (Tsukioka *et al*., 1997; Nakano and Ooshima, 2009). *Sm*RmlD and GacA are 82% identical (234 out of 284 residues), suggesting that they catalyze the same enzymatic reaction. We constructed a *S. mutans rmlD* mutant strain (SMUΔ*rmlD*) by replacing *rmlD* in frame with an erythromycin resistance cassette. Similar to disruption of *gacA* in GAS, loss of RmlD significantly affected cell morphology (Fig. 6A), bacterial growth (Fig. 6B) and resulted in complete loss of rhamnose (Fig. 6C) as previously published (Tsukioka *et al*., 1997). We next complemented SMUΔ*rmlD* with GAS *gacA* on a complementation plasmid (SMUΔ*rmlD* + p*GacA*). Expression of GacA restored *S. mutans* growth (Fig. 6B), rhamnose production (Fig. 6C) and almost completely restored cell appearance (Fig. 6A). These results demonstrate that *gacA* can functionally replace *rmlD* in *S. mutans*, supporting the role of GacA as a dTDP‐4‐dehydrorhamnose reductase.

## Conclusions

We have demonstrated through genetics, biochemistry, functional and structural analysis that GacA is the dTDP‐4‐dehydrorhamnose reductase RmlD homologue in GAS and is critical for normal growth. GacA represents the first structurally and biochemically characterized Gram‐positive RmlD homologue. In contrast to the published RmlD structure from Gram‐negative *S. enterica* (Blankenfeldt *et al*., 2002), GacA is representative of a new class of RmlD enzymes that are functional as a monomer. Bioinformatics analysis of 213 RmlD sequences complemented with experimental evidence from wild‐type and mutated *Se*RmlD, we have identified two sequence features that characterize the (putative) metal‐dependent dimeric RmlDs that are not conserved in monomeric RmlDs. Interestingly, dimeric RmlD enzymes cluster within Gram‐negative species, whereas monomeric RmlD enzymes are predominantly present in Gram‐positive bacteria. *Se*RmlD was described as a metal‐dependent enzyme as addition of EDTA reduced activity by about 70% (Graninger *et al*., 1999; Blankenfeldt *et al*., 2002). However, it remained unknown whether absence of Mg^2+^ results in loss of dimerization. It was speculated that the presence of Mg^2+^ stabilizes cofactor binding (Blankenfeldt *et al*., 2002). Our data show that the EDTA treated *Se*RmlD wild‐type enzyme remains dimeric (Supp. Fig. S2A), suggesting that the removal of Mg^2+^‐ion disturbs the proper tertiary structure of each monomer and therefore inactivates the enzyme.

From targeted mutagenesis attempts and Tn‐Seq studies, we conclude that *gacA* is essential for growth of GAS in rich medium, in agreement to a previous study that suggested that *gacA* might be essential (van Sorge *et al*., 2014). We validated that *gacA* is essential by a riboswitch‐inducible expression system resulting in attenuated growth and severe cellular abnormalities in the absence of *gacA*. Additional experiments in *S. mutans* confirm the critical role of GacA in rhamnose biosynthesis as *gacA* could functionally replace *rmlD* in *S. mutans* resulting in restored rhamnose in cell wall, growth and morphology.

In this study, we have identified GacA as an attractive drug target for the development of novel antimicrobial compounds against GAS. More importantly, these inhibitors could serve as lead compounds to inhibit L‐rhamnose biosynthesis in other bacteria. The dTDP‐L‐rhamnose biosynthesis is an interesting target for the development of new drugs since (i) the pathway affects either the viability or virulence of many bacteria, including *Mycobacterium spp.* (Ma *et al*., 2002), *Pseudomonas spp.* (Engels *et al*., 1985) and *E. faecalis* (Teng *et al*., 2009) and (ii) the pathway does not exist in humans, reducing the risk of side‐effects by off‐target effects. Known *Mtb*RmlD inhibitors inhibit recombinant GAS GacA in the mid‐ to low micromolar range in kinetic assays. However, we were unable to demonstrate a reliable effect of these compounds on GAS growth as the compounds are highly water insoluble. This implies that these compounds are not suitable as a starting point for structure‐based drug design on the basis of their chemical properties. The genetic, biochemical and structural data presented here on GAS GacA forms the framework for future screenings to identify novel inhibitors that target GAC biosynthesis in GAS and dTDP‐L‐rhamnose biosynthesis through related RmlD enzymes in other human pathogens.

## Experimental procedures

### GacA cloning, expression and purification

Full‐length *gacA* (accession number AAZ51220.1; M5005_Spy_0602) was PCR amplified from GAS genomic DNA (M1T1 5448) and cloned by restriction‐free cloning into a modified pGEX vector, with an N‐terminal hexa‐histidine‐GST‐tag followed by a PreScission Protease cleavage site (His_6_GST‐GacA). His_6_GST‐GacA was transformed in *E. coli* BL21(DE3) cells and recombinant protein expression was induced with 0.5 mM isopropyl‐1‐thio‐β‐D‐galactopyranoside for 18 h. Cells were harvested by centrifugation and resuspended in buffer A (50 mM Tris‐base, pH 8.0, 250 mM NaCl), supplemented with 10% glycerol, 0.5 complete Protease Inhibitor Cocktail Tablets (Roche) and 2 mM Tris (2‐Carboxyethyl)‐phosphine hydrochloride (TCEP). All purification steps were carried out at 4°C. Cells were disrupted, and the supernatant from two subsequent centrifugation runs at 15 000× *g* for 20 min and 100 000× *g* for 1.5 h was adjusted to 10 mM imidazole. The sample was passed over a 5 ml His‐trap column HP charged with Co^2+^, washed with 10 column volumes (CV) of 25 mM imidazole in buffer A, and the protein was eluted with a gradient over 10 CV of 500 mM imidazole in buffer A. The eluted protein was concentrated using a 50 k Da MW cutoff concentrator and passed over a desalting column, equilibrated in buffer A. The His_6_GST‐tag was cleaved using PreScission Protease overnight, passed over a 5 ml His‐trap column equilibrated in buffer A supplemented with 20 mM imidazole, the flow through was collected and concentrated using a 10 k Da MW cutoff concentrator and injected into a Superdex 75 26/60 column equilibrated in TBS‐buffer, supplemented with 0.2 mM TCEP. The fractions containing GacA were collected and concentrated to 25 mg ml^−1^. The purified protein was confirmed by tryptic fingerprint mass spec (University of St. Andrews).

### Enzymatic activity of GacA

To analyze GacA enzyme kinetics, we cloned and expressed GAS homologues of *rmlB* (*Sp*RmlB; accession number AAZ51354.1; M5005_Spy_0736) and *rmlC* (*Sp*RmlC; accession number AAZ51353.1; M5005_Spy_0735) using a modified pET vector, harboring an N‐terminal octa‐histidine tag. The RmlB and RmlC‐fusion proteins were expressed and purified as described for GacA. The assay was performed following the protocol from Sivendran *et al*. (2010) with the following changes: the assay buffer system contained 25 mM Tris‐base (pH 7.5), 150 mM NaCl, 0.1 mM NADPH and 2 pM GacA. *Sp*RmlB and *Sp*RmlC were added to the assay in 25‐fold molar excess relative to GacA. The assay was started with the addition of 400 μM dTDP‐D‐glucose. Michaelis‐Menten kinetics for GacA for dTDP‐glucose was calculated using a concentration range from 0.025 to 1 mM of dTDP‐glucose. The oxidation of NADPH to NADP^+^ was measured by the change in intrinsic absorbance at 340 nm using a SpectraMax M2 plate reader. Background absorbance was subtracted and data interpreted using the Michaelis‐Menten model in GraphPad Prism (GraphPad Software, Inc., 7825 Fay Avenue, Suite 230, La Jolla, CA 92037 USA). RmlD inhibitors 2 and 3 (Sigma) described for *M. tuberculosis* (Wang *et al*., 2011) were dissolved to 10 mM stock concentration in 100% DMSO, diluted in enzyme assay buffer and added at the same time‐point as the GacA enzyme. All reactions contained a final concentration of 2% DMSO.

### SeRmlD cloning, expression and purification

Full‐length *rmlD* (GI 16420628) was PCR amplified from *S. enterica* genomic DNA (LT2) using the procedure described for *gacA*. Point mutations were inserted using standard mutagenesis procedures. Wild type and mutant enzymes were expressed and purified as described for GacA. EDTA treatment was performed by incubation of *Se*RmlD (0.1 mg ml^−1^) with 10 mM EDTA for 30 min at room temperature, followed by a concentration‐step and size‐exclusion chromatography in buffer A containing 10 mM EDTA.

### Surface plasmon resonance (SPR) experiments

Recombinant, purified GacA was chemically biotinylated and captured on a streptavidin surface of a Biacore T200 instrument (GE‐Healthcare) at densities of 3 k–4 k RU. To stabilize captured protein over time, all experiments were run at 10°C. Ligands were injected over captured protein at flow rate 30 μl min^−1^ in running buffer (50 mM Tris, pH 7.4, 150 mM NaCl, 0.05%Tween, 3% DMSO), with each compound injected in duplicates in concentration series 4–1000 μM. Association was measured for 60 s and dissociation for 120 s. All data were double referenced for blank injections of buffer and biotin‐blocked Streptavidin surface. Scrubber 2 (BioLogic Software) was used to process and analyze the data.

### Crystallization and structure determination

Vapor diffusion sitting‐drop crystallization was carried out at 20°C. Crystals appeared after mixing equal amounts of protein and crystallization buffer containing 12.5% w/v PEG 1000, 12.5% w/v PEG 3350, 12.5% v/v MPD, 0.02 M of each carboxylic acid (0.2 M sodium formate, 0.2 M ammonium acetate, 0.2 M trisodium citrate, 0.2 M sodium potassium L‐tartrate, 0.2 M sodium oxamate) and 0.1 M MES/imidazole pH 6.5 (Gorrec, 2009) after 1 day. Crystals were flash frozen into liquid nitrogen prior to data collection. Data were collected at beamline ID23‐1 at ERSF and processed with iMOSFLM (Battye *et al*., 2011). Data collection statistics are summarized in Table 3. On the basis of the high‐resolution dataset, the structure was solved *ab initio* using the program ‘Acorn’ in the CCP4 suite (McCoy *et al*., 2007). The initial model was used for autobuilding in Phenix (Adams *et al*., 2010). With the exception of the first and last residue, 288 residues out of 290 residues were built, and the structural model was refined in iterative cycles using Coot (LMB Cambridge, UK) (Emsley *et al*., 2010) and Refmac (Murshudov *et al*., 1997) to the statistics shown in Table 3. The final model was refined to 1.1 Å resolution with an R_factor_ of 12.9% and R_free_ of 15.5%. The Ramachandran plot revealed that 98% of all residues are in favored regions, with no outliers as calculated by MolProbity (Lovell *et al*., 2003). The co‐ordinates and structure factors have been deposited with the RCSB Protein Data Bank with PDB ID code 4WPG.

**Table 3:** Crystallographic data collection and refinement statistics

Spacegroup	P1
Unit cell dimension (Å)	36.48 45.98 48.87
Resolution range (last shell) (Å)	43.8–1.1 (1.139–1.1)
Unit cell angles (°)	66.3 81.21 95.27
Unique reflections	108172 (9823)
Completeness (%)	94.0 (85.5)
I/σI	22.5 (5.7)
Wilson B‐factor	12.20
R_factor_	0.129 (0.175)
R_free_	0.155 (0.205)
Number of atoms	5062
Macromolecules	2314
Ligands	21
Water	468
Protein residues	287
Rms bond length (Å)	0.023
Rms bond angles (°)	2.08
Ramachandran favored (%)	98
Ramachandran outliers (%)	0
Average B‐factor	18.10
Macromolecules	16.10
Solvent	27.90

I/σI, intensity divided by standard deviation of intensity, averaged over all measurement.

Rms is root‐mean‐square deviation from ideal value.

### Neighbor‐Joining tree construction

To construct midpoint‐rooted Neighbor‐Joining trees of RmlD sequences, we used two‐independent psi‐BLAST runs using *Se*RmlD and GacA as the query sequences. We obtained 135 Gram‐negative RmlD homologues of *Se*RmlD using an E‐value cutoff of > 7e^−58^, and we obtained 78 Gram‐positive homologues of GAS GacA using a cutoff of > 1e^−48^. All hypothetical sequences and all RmlD sequences for which no corresponding 16S rRNA sequences were available were rejected. We used only one representative for each bacterium, avoiding the use of multiple strain variants. Multiple sequence alignments of RmlD protein sequences and 16S rRNA sequences were constructed using ClustalW2 (McWilliam *et al*., 2013). Neighbor‐Joining trees were built from the RmlD and 16S rRNA alignments using ClustalX (2000 bootstraps were run) (Larkin *et al*., 2007). Neighbor‐Joining trees were displayed using FigTree (http://tree.bio.ed.ac.uk/software/figtree/). All bacteria analyzed were assigned to the corresponding monomer/dimerization motif groups and were marked with related colors.

### Tn‐seq to identify genes essential for GAS growth in THY

The pKRMIT plasmid contains a *mariner* mini‐transposon named *Krmit* (Kanamycin‐resistant element for massive identification of transposants) modified for Tn‐seq (Le Breton *et al*., 2015) and was used to perform saturating transposition for random mutagenesis in GAS 5448 and NZ131 as previously described for the *mariner* transposon *Oskar* (Le Breton and McIver, 2013; Le Breton *et al*., 2013). Tn‐seq (van Opijnen and Camilli, 2010) was performed as described recently (Le Breton *et al*., 2015) with the following primers oKrmit‐Tnseq2 (5′‐CAAGCAGAAGACGGCATACGAAGCGCCTACG‐AGGAATTTGTATCG‐3′) and oAdapterPCR (5′‐ACACTCTTTCCCTACACGACGCTCTT‐CCGATCT‐3′), resulting in the production of 176 bp *Krmit* insertion tags. Quality and yield of the resulting tags was assessed using a NanoDrop spectrophotometer (Thermo Scientific) and an Agilent Bioanalyzer. *Krmit* insertion tags were analyzed by Illumina sequencing (50 nt single end reads) on a HiSeq 1500 platform in the Institute for Bioscience and Biotechnology Research (IBBR) Sequencing Facility located at the University of Maryland, College Park. The quality of read datasets (Sanger FastQ format) was determined using FastQC (Ramirez‐Gonzalez *et al*., 2013); data were filtered and trimmed using Biopieces (biopieces.org) to select for reads containing the Tn‐seq barcodes and *Krmit* ITR ends. Reads were then de‐multiplexed and count tables generated using SamTools (Li *et al*., 2009) and HTseq (Chandramohan *et al*., 2013). Reads were mapped to the GAS 5448 or NZ131 genome using Bowtie (Langmead *et al*., 2009) and data relevant to the *gacA‐L* locus visualized using the Integrative Genomics Viewer (IGV) browser (broadinstitute.org/igv/home). Gene essentiality was determined using a Bayesian statistical model based on the Metropolis‐Hastings algorithm using the Python script (saclab.tamu.edu/essentiality) developed by DeJesus *et al*. (2013).

### Construction of a conditionally lethal gacA mutant

A GAS 5448 merodiploid mutant, gacAi, was constructed using the pSinS/pHplK system for gacA gene expression to be under the control of a theophylline‐dependent riboswitch (Topp *et al*., 2010) as recently described (Le Breton *et al*., 2015). Succinctly, a ca. 600‐nu fragment of the 5′‐end of the gacA gene was amplified using primers oGacA‐1 (5′‐CCCTGCTAAGGAGGTAACAACAAGATGATTTTAATTACAGGAAGCAATGG‐3′) and oGacA‐2 (5′‐cccGGATCCGTCAAATAACACATGAATTCTGC‐3′) and subsequently fused by SOE‐PCR to the Psag promoter along with the synthetic riboswitch E as previously described (Le Breton *et al*., 2015). The resulting PCR product was then cloned into the BamHI site of the pSinS plasmid producing pGacAi and mutation carried out as described (Le Breton *et al*., 2015), creating the GAS 5448 gacAi mutant; and the junction between the Psag promoter, the synthetic riboswitch E and the gacA gene in the GAS 5448 gacAi mutant was verified by DNA sequencing (data not shown).

### Bacterial strains and growth conditions

*Streptococcus mutans* Xc is a serotype C wild‐type strain (Koga *et al*., 1989) and was kindly provided by Dr. Y Yamashita (Kyushu University, Japan). *S. mutans* was routinely cultured in Todd‐Hewitt Broth (THB; Oxoid) or on THB agar at 37°C with 5% CO_2_. GAS strain 5448 is a representative of the serotype M1T1 clone (Kansal *et al*., 2000) and GAS NZ131 (Simon and Ferretti, 1991) is an invasive strain of the M49 serotype. GAS was grown in THB (Becton Dickinson) supplemented with 1% yeast extract (THY) or on THY agar at 37°C. When required, growth medium was supplemented with 10 μg ml^−1^ erythromycin (Erm) or 3 μg ml^−1^ chloramphenicol (Cm) for *S. mutans* or with 300 μg ml^−1^ kanamycin (Km) or 100 μg ml^−1^ spectinomycin (Spec) for GAS. For cloning purposes, *E. coli* strain MC1061 was grown in Luria–Bertani (LB; Oxoid) or on LB agar with ampicillin (100 μg ml^−1^, Amp), Erm (500 μg ml^−1^) or Cm 10 μg ml^−1^.

### Genetic manipulation of S. mutans

To confirm the function of GacA in the production of dTDP‐L‐rhamnose in bacteria, we heterologously expressed GAS *gacA* (M5005_Spy_0602; accession number AAZ51220.1) in a *S. mutans rmlD* deletion mutant (SMUΔ*rmlD*), which is unable to produce dTDP‐L‐rhamnose (Tsukioka *et al*., 1997). For complementation, full‐length *gacA* was amplified from the GAS 5448 chromosome using primers XbaI_*gacA*F 5′‐GCTCTAGAATGATTTTAATTACAGGAAGCAATGGTC‐3′ and BamHI_*gacA*R 5′‐CGCGGATCCTACTTACTTTTTCAGTCCTTGTTGGT‐3′ and cloned into expression vector pDC123 using XbaI and BamHI restriction sites, yielding p*GacA*. p*GacA* was transformed into *S. mutans* wild‐type and selected for Cm resistance. The presence of p*GacA* was confirmed by PCR analysis. Subsequently, *rmlD* was knocked out by precise in frame allelic replacement of *rmlD* with an Erm resistance gene in *S. mutans* + p*GacA*. Briefly, 700 bp immediately upstream of *rmlD* was amplified with the primers *rmlD*upF, 5′‐CGCAGCAAGCAGTTACGTGATTTTGTTGAAG‐3′, and *rmlD*upR+erm 5′‐GTTTTGAGAATATTTTATATTTTTGTTCATTATTTTTTCTCCTTTAAAAAGCTTTATTACTATTACC‐3′, and 674 bp immediately downstream of *rmlD* was amplified with the primers *rmlD*downF+erm 5′‐AGTTATCTATTATTTAACGGGAGGAAATAATATTTTAGCAAA‐GAAGGACAGGTTTAAACC‐3′, and *rmlD*downR, 5′‐CTGAAGGTGATAA‐ATCCGTGCCATA‐3′. The *rmlD*upR+erm and *rmlD*downF+erm primers were constructed with 30 bp 5′ extensions (underlined) corresponding to the 5′ and 3′ ends of the *erm* gene respectively. The upstream and downstream PCR fragments were combined with the 738 bp amplicon of the *erm* gene [amplified off the pDCerm plasmid (Jeng *et al*., 2003)] as templates in a second round of PCR using primers *rmlD*upF and *rmlD*downR. The resultant PCR amplicon, containing an in frame substitution of *rmlD* with *erm*, was transformed into *S. mutans* + p*GacA* and selected for Erm and Cm resistance as previously described (Perry *et al*., 1983), to yield *S. mutans* Δ*rmlD* + p*GacA* (SMUΔ*rmlD* + p*GacA*). PCR analysis was used to confirm the deletion of *rmlD*.

### Scanning Electron Microscopy (SEM)

Overnight cultures of *S. mutans* strains were diluted and grown to mid‐log phase (*S. mutans* wild‐type and *S. mutans* Δ*rmlD* + p*GacA* OD_600_ of 0.3, *S. mutans* Δ*rmlD* OD_600_ of 0.15). GAS WT strains were grown in THY medium overnight. GAS 5448 gacAi were selected from a plate containing Spec and 2 mM theophylline and grown overnight cultures in the presence of different concentrations of theophylline (0, 0.5, 1, 1.5 and 2 mM) in THY. All cultures reached an OD_600_ of 0.5/0.6 except for strains without theophylline, which reached OD_600_ of 0.35. Cultures were diluted in THY with or without theophylline to an OD_600_ of 0.06 and grown to midlog phase. Samples were washed, fixed and dehydrated as described previously (Garufi *et al*., 2012), mounted onto 12.5 mm specimen stubs (Agar scientific, Stansted, Essex, UK) and coated with gold to 1 nm using a Quorum Q150R S sputter coater at 20 mA. Visual examination was performed with a Phenom PRO desktop SEM (Phenom‐World BV). The SEM was operated with an acceleration voltage of 10 kV.

### S. mutans growth curves

Growth curves of *S. mutans* Xc wild‐type, Δ *rmlD* and Δ *rmlD* + p*GacA* were obtained after dilution of an overnight culture to OD_600_ to 0.025 in THB. Optical density was recorded every 30 min over 12 h at 37°C without 5% CO_2_ in a 100 Honeycombe plate using a Bioscreen C MBR machine (Growth Curves AB Ltd, Oy, Finnland).

### Carbohydrate analysis of S. mutans strains

For the isolation of cell wall carbohydrates in *S. mutans*, 2 l bacterial cultures were centrifuged (8000× g, 30 min, 4°C) and washed with ice‐cold water. Five grams of wet bacterial cells was resuspended in 0.1 M citrate buffer (pH 4.6, 4°C) and disrupted with a bead‐beater (Biospec). The bacterial lysate was centrifuged (1000×  g, 5 min, 4°C), the white suspension was isolated and centrifuged (35 000× g, 30 min, 4°C) to collect the cell walls. The white pellet was washed with citrate buffer, resuspended in 0.1 M sodium acetate (pH 4.6) containing 4% sodium dodecyl sulfate (SDS) and boiled for 1 h at 100°C with agitation. The suspension was centrifuged (35 000× g, 30 min, 21°C) and washed with SuperQ to remove SDS. The pellet was resuspended in 10 ml MilliQ and treated with RNase (10 μg ml^−1^) and DNase (10 μg ml^−1^, 37°C, 2 h) and afterward with pronase E (100 μg ml^−1^, 37°C for 24 h). Cell wall carbohydrates were extensively washed with SuperQ (35 000× g for 30 min), treated with trypsin (100 μg ml^−1^, 37°C, 2 h), washed with SuperQ and lyophilized before the sample was subjected to hydrolysis with TFA according to published procedures (Fan *et al*., 1994). Carbohydrate analysis was performed on a Dionex ICS‐3000 Ion Chromatography System (Dionex / Thermo Scientific, 1228 Titan Way, P.O. Box 3603, Sunnyvale, CA, 94088‐3603, United States) using a CarboPac PA20 (Dionex / Thermo Scientific, 1228 Titan Way, P.O. Box 3603, Sunnyvale, CA, 94088‐3603, United States) 3 × 150 mm column, equipped with a ICS‐3000 Electrochemical Detector (Dionex / Thermo Scientific, 1228 Titan Way, P.O. Box 3603, Sunnyvale, CA, 94088‐3603, United States). The ‘Carbohydrates (Standard Quad)’ waveform was used for detection. Eluent was run at 92% H_2_O and 8% 0.2M NaOH(aq) for 25 min and increased to 100% 0.2 M NaOH over a 2 min period and held at this concentration for 10 min. The concentration was then returned to 92% H_2_O and 8% 0.2 M NaOH(aq) over a 2 min period and held at this concentration for 11 min before a new sample was injected. The flow rate was 0.5 ml min^−1^.

## Supporting information

Supporting information
