# Supplementary material for: GacA is essential for Group A S treptococcus and defines a new class of monomeric dTDP‐4‐dehydrorhamnose reductases (RmlD)
Source: Mol Microbiol. 2015 Oct 1;98(5):946–62. doi: 10.1111/mmi.13169 (PMC4832382; doi:10.1111/mmi.13169)
Supplement: Supplementary file 1 — Supporting information [file MMI-98-946-s001.zip › MMI_13169_Supplement_proof_combined.pdf]

Supplementary Information

GacA is Essential for Group A *Streptococcus* and Defines a New Class of Monomeric dTDP-4-dehydrorhamnose Reductases (RmlD)

Samantha L. van der Beek[a], Yoann Le Breton[b], Andrew T. Ferenbach[c], Robert N. Chapman[d], Daan M.F. van Aalten[c], Iva Navratilova[e], Geert-Jan Boons[d], Kevin S. McIver[b], Nina M. van Sorge[a, *] and Helge C. Dorfmueller[c,f,*]

[a]University Medical Center Utrecht, Medical Microbiology, Heidelberglaan 100, 3584 CX Utrecht, The Netherlands
[b]Department of Cell Biology and Molecular Genetics, Maryland Pathogen Research Institute, University of Maryland, 3124 Biosciences Research Building, College Park, MD 20742, United States of America
[c]Division of Molecular Microbiology, University of Dundee, School of Life Sciences, Dow Street, DD1 5EH, Dundee, United Kingdom
[d]Complex Carbohydrate Research Center, Department of Chemistry, The University of Georgia, 315 Riverbend Road, Athens, USA
[e]Division of Biological Chemistry and Drug Discovery, University of Dundee, School of Life Sciences, Dow Street, DD1 5EH, Dundee, United Kingdom
[f]Rutherford Appleton Laboratory, Research Complex at Harwell, Didcot OX11 0FA, United Kingdom

*Correspondence:
n.vansorge-3@umcutrecht.nl, phone: +31 (0) 88 75 57627 and
hczdorfmueller@dundee.ac.uk, phone: +44 (0) 1382 386302

Keywords: Carbohydrate biosynthesis, *Streptococcus pyogenes*, enzyme structure, structural biology, short chain reductase, L-rhamnose

Running title: GacA is an essential monomeric RmlD enzyme

SUPPLEMENT FIG. 1

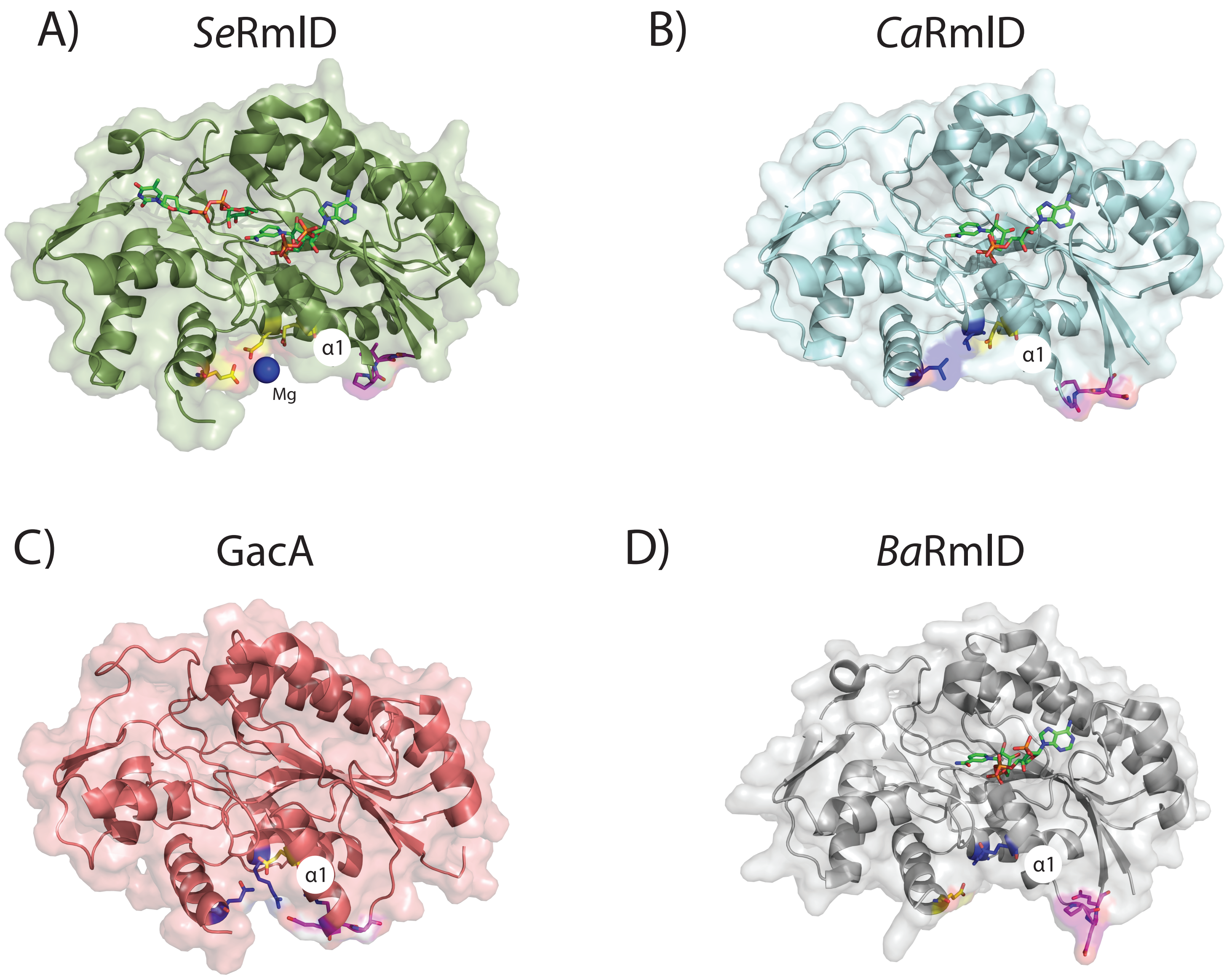

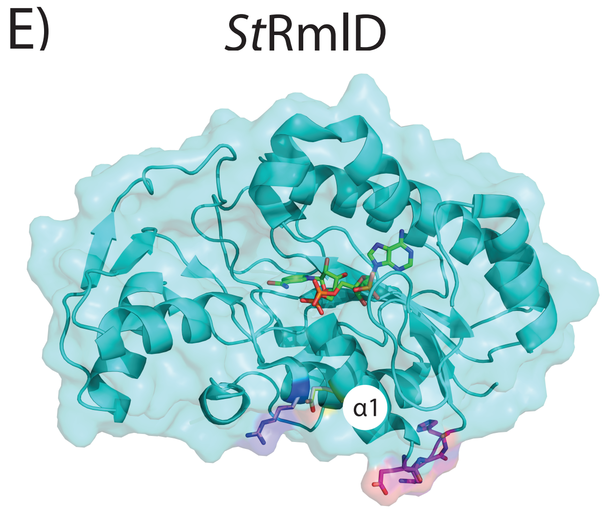

Legend:
*Se*RmlD, GacA and putative RmlD enzymes shown in surface representation (transparent) with secondary structure elements (cartoon). Residues of the sequence logo (Fig. 4C) are shown in sticks with red oxygen atoms and blue nitrogen atoms. Mg-binding site residues are shown in yellow, non-conserved corresponding residues in blue, according to Fig. 1. Residues of the α1-helix motif are shown in magenta. A) One monomer is shown of the Gram-negative *Se*RmlD homo-dimer (1KC3.pdb) ternary complex with NADPH and dTDP-glucose (green sticks). The Gram-positive orthologues are shown in B-D), with B) *Ca*RmlD (1VL0.pdb), C) GacA and D) *Ba*RmlD (2SC6.pdb). E) Structure of the putative RmlD orthologue from the archaea *Sulfolobus tokodaii* RmlD (*St*RmlD, 2GGS.pdb). *St*RmlD shares sequence conservation with the Gram-positive motifs. The Gram-positive and archaea RmlD homologues do not form crystallographic dimers and do not possess a Mg-binding site. These sequence features are accommodated by large residues at the end of the α1-helix.

SUPPLEMENT FIG. 2

A)

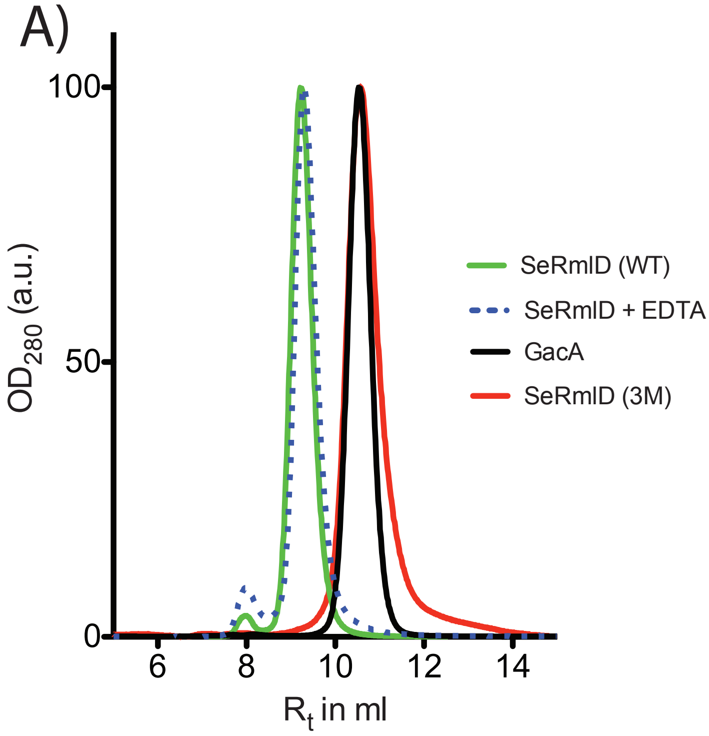

Legend:

A) Purified GacA, *Se*RmlD wild type (WT) and *Se*RmlD mutant (3M) were analysed on a Superdex 75 size exclusion column. The *Se*RmlD WT shows a retention volume of 9.3 ml, identical to a EDTA treated sample (*Se*RmlD + EDTA). GacA of 10.7 ml, suggesting that *Se*RmlD forms a dimer. This data is in agreement with the literature (Blankenfeldt *et al.*). The *Se*RmlD triple mutant runs at a retention volume of 10.7 ml, identical with GacA, suggesting that the *Se*RmlD dimer has been disrupted into monomers, by mutating the residues at the dimer interface and replacing them with the GacA residues.
