# Supplementary material for: GacA is essential for Group A S treptococcus and defines a new class of monomeric dTDP‐4‐dehydrorhamnose reductases (RmlD)
Source: Mol Microbiol. 2015 Oct 1;98(5):946–62. doi: 10.1111/mmi.13169 (PMC4832382; doi:10.1111/mmi.13169)
Supplement: Supplementary file 1 — Supporting information [file MMI-98-946-s001.zip › MMI_13169_tables S1-S3.docx]

**Supplement Tables**

**Supp. Tab. 1**

Gram-negative bacteria (in alphabetical order) that contain a RmlD homologue with the sequence motifs E1, α1-helix, E2 and E3. These 70 sequences were used to create the sequence logo 'Gram-negative 1' for dimeric RmlD enzymes (Fig. 4C).

*Acidovorax_sp. E-PLG-E-E*

*Acinetobacter_guillouiae E-PLG-A-E*

*Aeromonas_hydrophila E-PLG-E-E*

*Alcanivorax_borkumensis E-PFG-E-E*

*Alicycliphilus_denitrificans E-VLG-D-E*

*Alkanindiges_illinoisensis E-PLG-A-E*

*Alkalilimnicola_ehrlichii E-PLG-E-E*

*Aromatoleum_aromaticum E-PLG-R-E*

*Azoarcus_sp. E-PLG-D-E*

*Azohydromonas_australica E-VLG-D-E*

*Azotobacter_chroococcum E-PLG-E-E*

*Buttiauxella_agrestis E-PLG-E-E*

*Candidatus_accumulibacter_phos E-PLG-E-E*

*Cedecea_neteri E-PLG-E-E*

*Citrobacter_freundii E-PLG-E-E*

*Comamonas_composti E-PLG-D-E*

*Cronobacter_pulveris E-PLG-E-E*

*Curvibacter_lanceolatus E-PLG-D-E*

*Delftia_sp. E-PLG-D-E*

*Desulfobacter_postgatei E-PLG-E-E*

*Desulfococcus_multivorans E-PLG-E-E*

*Edwardsiella_tarda E-PLG-E-E*

*Enterobacter_asburiae E-PLG-E-E*

*Erwinia_mallotivora E-PLG-E-E*

*Escherichia_coli E-PLG-E-E*

*Gallionella_capsiferriformans E-PLG-E-E*

*Gamma_proteobacterium_HdN1 E-PLG-E-E*

*Hafnia_paralvei E-PLG-E-E*

*Halomonas_salina E-PLG-E-E*

*Herbaspirillum_frisingense E-PLG-E-E*

*Hylemonella_gracilis E-PLG-D-E*

*Ideonella_sp. E-PLG-E-E*

*Klebsiella_pneumoniae E-TLG-E-E*

*Kosakonia_radicincitans E-PLG-E-E*

*Leclercia_adecarboxylata E-PLG-E-E*

*Leminorella_grimontii E-PLG-E-E*

*Leptothrix_cholodnii E-PLG-D-E*

*Limnohabitans_sp. E-PVG-D-E*

*Marinobacter_hydrocarbonoclast E-VLG-E-E*

*Methylibium E-PLG-D-E*

*Methylobacter_tundripaludum E-PLG-D-E*

*Methyloglobulus_morosus E-PLG-R-E*

*Methylomarinum_vadi E-PLG-R-E*

*Methylomonas_methanica E-PLG-E-E*

*Methylotenera_versatilis E-PLG-D-E*

*Methyloversatilis_universalis E-PLG-E-E*

*Morganella_morganii E-PLG-E-E*

*Nitrosomonas_sp. E-PLG-E-E*

*Nitrosospira_briensis E-PLG-D-E*

*Ottowia_thiooxydans E-PLG-D-E*

*Pantoea_rwandensis E-PLG-E-E*

*Polaromonas_naphthalenivorans E-PLG-E-E*

*Providencia_alcalifaciens E-PLG-E-E*

*Pseudomonas_tolaasii E-PLG-D-E*

*Rahnella_aquatilis E-PLG-E-E*

*Ramlibacter_tataouinensis E-PLG-D-E*

*Raoultella_planticola E-TLG-E-E*

*Rubrivivax_benzoatilyticus E-PLG-D-E*

*Salmonella_enterica E-PVG-E-E*

*Serratia_marcescens E-PLG-E-E*

*Shigella_dysenteriae E-PLG-E-E*

*Simplicispira_psychrophila E-VLG-D-E*

*Stenoxybacter_acetivorans A-PLG-D-D*

*Tatlockia_micdadei E-PLG-D-E*

*Thauera_linaloolentis E-PLG-D-E*

*Thiomonas_sp. E-PLG-E-E*

*Uliginosibacterium_gangwonense E-PLG-E-E*

*Variovorax_paradoxus E-PLG-E-E*

*Vibrio_rotiferianus E-CLG-E-E*

*Xenorhabdus_bovienii E-PLG-E-E*

**Supp. Tab. 2**

Sequence motif of 65 Gram-negative bacteria (in alphabetical order) that lack the dimerization motifs. These motifs were used to determine the 'Gram-negative 2' RmlD sequence logo (Fig. 4C).

*Achromobacter_piechaudii E-PLG-E-Q*

*Advenella_kashmirensis A-ASA-R--*

*Aequorivita_sublithincola S-NTE-G-N*

*Alkaliflexus_imshenetskii T-SSQ-T-N*

*Alteromonas_macleodii E-NSE-K-E*

*Andreprevotia_chitinilytica E-VLG-S-Q*

*Aquaspirillum_serpens E-CVG-S-S*

*Arenibacter_certesii S-YPE-G--*

*Arenimonas_oryziterrae E-PLG-R-E*

*Asticcacaulis_excentricus S-ALG-P-R*

*Azospirillum_brasilense E-PGL-A-D*

*Burkholderia_ambifaria E-PLG-R-E*

*Cellulophaga_algicola C-RDY-E-S*

*Cellvibrio_mixtus E-CIG-A-E*

*Chitinimonas_koreensis E-GLG-R-D*

*Chlorobium_ferrooxidans E-LTG-A-E*

*Chlorogloeopsis_fritschii E-PYG-R-E*

*Chromobacterium_piscinae E-PLG-A-A*

*Coprobacter_fastidiosus N-EED-G-L*

*Cupriavidus_sp. E-PLG-A-Q*

*Dechloromonas_aromatica Q-PHG-A-L*

*Deefgea_rivuli E-VLG-S-L*

*Derxia_gummosa E-PLG-R-D*

*Desulfobulbus_propionicus E-PLG-S-Q*

*Desulfocapsa_sulfexigens D-ADH-A-L*

*Dysgonomonas_capnocytophagoi E-HND-A-E*

*Ectothiorhodospira_sp. E-PLG-R-E*

*Edaphobacter_aggregans E-PLG-R-E*

*Flavobacterium_cauense A-HPD-V-V*

*Formosa_agariphila C-HNN-T-N*

*Gluconobacter_oxydans S-EHI-D-K*

*Hyphomicrobium_sp. S-ASG-L-R*

*Inquilinus_limosus E-QGL-A-T*

*Jannaschia_sp. E-TLG-R-D*

*Labrenzia_aggregata E-EMG-A-H*

*Leeia_oryzae E-LLG-A-L*

*Legionella_pneumophila E-SEY-R-H*

*Mariprofundus_ferrooxydans E-QGH-A-E*

*Methylocystis_sp. S-RGV-L-R*

*Microbulbifer_agarilyticus E-PED-R-E*

*Neisseria_meningitidis C-EDW-G-K*

*Nevskia_soli E-PLG-H--*

*Oceanimonas_sp. E-PLG-A-V*

*Opitutus_terrae E-PMS-R-S*

*Paludibacterium_yongneupense E-PLG-A-S*

*Parabacteroides_merdae S-YPQ-G-G*

*Paracoccus_pantotrophus E-PWA-A-E*

*Pectobacterium_carotovorum C-PAE-G--*

*Pelodictyon_luteolum E-GAG-A-T*

*Planktothrix_agardhii E-SLG-G-E*

*Prevotella_copri H-QKS-G-E*

*Pseudoxanthomonas_suwonensis E-PLG-A-Q*

*Ralstonia_pickettii E-TLG-S-Q*

*Reinekea_blandensis E-VFG-R-E*

*Rheinheimera_texasensis C-RLG-G--*

*Rhizobium_leguminosarum A-GAI-N-K*

*Rhodopseudomonas_palustris S-ADI-L-R*

*Rubellimicrobium_thermophilum Q-ALG-D-E*

*Sideroxydans_lithotrophicus E-AFG-K-L*

*Silanimonas_lenta E-ARG-H-Q*

*Stenotrophomonas_maltophilia E-PLG-A-D*

*Sulfuricella_denitrificans E-TLG-R-K*

*Tannerella_forsythia S-NHH-G-T*

*Thiobacillus_denitrificans E-PLG-R-Q*

*Verminephrobacter_eiseniae Q-VLG-E-E*

**Supp. Tab. 3**

Seventy-eight Gram-positive bacteria (in alphabetical order) that contain an RmlD/GacA homologues. The table shows the corresponding sequence for motifs X1, α1-helix, X2 and X3, which were used to create the 'Gram-positive' sequence logo (Fig. 4C).

*Acidaminococcus_fermentans D-KRG-A-E*

*Actinobaculum_massiliense D-REG-C-S*

*Acidothermus_cellulolyticus D-RSG-R-F*

*Aerococcus_viridans D-EKK-Y-E*

*Anaeroarcus_burkinensis D-SRR-A-E*

*Anaerostipes_caccae D-KRG-Y-E*

*Anaerotruncus_colihominis D-RRG-A-E*

*Anaerovibrio_lipolyticus D-KRG-Y-E*

*Aneurinibacillus_terranovensis D-QNR-V-E*

*Anoxybacillus_tepidamans E-QTN-V-T*

*Atopobacter_phocae E-DQA-K--*

*Atopococcus_tabaci E-ENG-R-S*

*Bacillus_anthracis Q-PEE-A-E*

*Bacteroides_pectinophilus D-KRG-P-E*

*Blautia_hydrogenotrophica D-KRG-Y-I*

*Butyrate-producing_bacterium D-KRG-Y-E*

*Caldanaerobacter_subterraneus Q-EKG-E-D*

*Caldicellulosiruptor_bescii E-NKY-K-L*

*Candidatus_Stoquefichus_massi D-KRN-Y-I*

*Carnobacterium_alterfunditum E-EKE-R-S*

*Clostridium_acetobutylicum E-GKN-V-L*

*Cohnella_panacarvi E-KRC-H-R*

*Coprococcus_comes D-NRG-A-E*

*Desulfitibacter_alkalitoleran E-TLG-R-E*

*Desulfosporosinus_meridiei D-RRG-L-E*

*Desulfotomaculum_kuznetsovii A-RRG-V-G*

*Dorea_longicatena D-SRG-F-E*

*Enterococcus_faecium E-EQG-R-N*

*Erysipelatoclostridium D-NRN-Y-E*

*Erysipelotrichaceae_bacterium D-KRG-L-E*

*Eubacterium_rectale D-ARG-L-E*

*Exiguobacterium_antarcticum D-SSL-E-K*

*Faecalibacterium_prausnitzii E-GPV-V-H*

*Fibrobacter_succinogenes D-KRG-L-E*

*Fictibacillus_gelatini D-DRG-H-E*

*Geobacillus_stearothermophilu D-DRG-V-R*

*Holdemanella_biformis D-SRG-Y-E*

*Jeotgalibacillus_campisalis D-KNG-F-Q*

*Kandleria_vitulina D-KRG-L-E*

*Knoellia_aerolata D-DAG-A-S*

*Lachnoanaerobaculum_sp. D-NRN-P-E*

*Lachnoclostridium_phytofermen D-KRN-Y-E*

*Lacticigenium_naphtae E-EME-R-I*

*Lactobacillus_brevis E-KRG-K-D*

*Lactococcus_lactis E-ERG-R-K*

*Leuconostoc_citreum E-ERD-R-S*

*Listeria_seeligeri E-EHN-Y--*

*Marvinbryantia_formatexigens D-KRG-Y-A*

*Megamonas D-ARN-V-E*

*Megasphaera_sp. D-KRG-L-V*

*Melissococcus_plutonius E-ELA-R-S*

*Mitsuokella_multacida D-DRG-R-E*

*Moorella_thermoacetica A-GRN-V-E*

*Mycobacterium_tuberculosis Y-SQG-G-A*

*Oenococcus_oeni E-DRG-K-I*

*Oribacterium_sp. D-RRG-L-E*

*Paenibacillus_borealis D-GQD-V-E*

*Pediococcus_acidilactici E-EKG-R-N*

*Pelosinus_fermentans E-GRG-V--*

*Peptoclostridium_difficile E-EKN-R-A*

*Peptostreptococcaceae_bacterium A-KSD-K-E*

*Pseudoramibacter_alactolyticu D-ARG-A-A*

*Roseburia_intestinales A-GED-V-T*

*Ruminococcus_obeum D-KRG-Y-E*

*Selenomonas_ruminantium D-SRG-R-E*

*Sharpea_azabuensis D-KRG-Y-E*

*Sporolactobacillus_laevolacti E-EKG-E-K*

*Stomatobaculum_longum D-RRG-L-L*

*Streptococcus_agalactiae E-ERT-R-Q*

*Streptococcus_dysgalactiae E-ERH-R-Q*

*Streptococcus_pyogenes (GAS) E-ERG-R-Q*

*Streptococcus_suis E-ERN-R-Q*

*Streptomyces_bicolor D-GRG-A-R*

*Thermicanus_aegyptius D-DRT-V-S*

*Thermoanaerobacter_wiegelii Q-KGR-E-D*

*Trueperella_pyogenes D-ERG-R-R*

*Veillonella_atypica D-EQH-E-I*

*Weissella_koreensis E-ERN-R-E*
